# Effects of uncomplicated Descemet membrane endothelial keratoplasty on the central retinal thickness

**DOI:** 10.1007/s00417-021-05203-2

**Published:** 2021-05-11

**Authors:** Tibor Lohmann, Sabine Baumgarten, Niklas Plange, Peter Walter, Matthias Fuest

**Affiliations:** Department of Ophthalmology, RWTH Aachen University, Pauwelsstrasse 30, 52074 Aachen, Germany

**Keywords:** Lamellar corneal surgery, Descemet membrane endothelial keratoplasty, Macular edema, Retinal thickness

## Abstract

**Purpose:**

To determine retinal thickness (RT) changes and the incidence of macular edema after uncomplicated Descemet membrane endothelial keratoplasty (DMEK-ME) in patients without ME risk factors.

**Methods:**

In this retrospective study, 107 pseudophakic eyes of 74 patients with Fuchs endothelial dystrophy (FED) (79.4%) or bullous keratopathy (BK) (20.6%) underwent DMEK surgery between 2016 and 2019 at the Department of Ophthalmology, RWTH Aachen University. Patients with intra- or postoperative complications as well as pre-existing risk factors for ME were excluded. Macular spectral-domain optical coherence tomography (SD-OCT) and best spectacle-corrected visual acuity (BSCVA) measurements were performed before, 1 week, 1 month, and 6 months after surgery. Retinal thickness (RT) was analyzed in the central foveal 1 mm (CSF), parafoveal 3 mm and 6 mm subfield.

**Results:**

Eight eyes (7.5%) developed DMEK-ME 1 month after surgery. Six DMEK-ME eyes (75%) were rebubbled, compared with 31.3% (31 of 99; *P* = 0.02) of the non DMEK-ME eyes. DMEK-ME eyes had a significantly thicker CSF 1 month after surgery (432.0 ± 97.6 μm) compared with non-DMEK-ME eyes (283.7 ± 22.2 μm; *P* = 0.01). The other subfields and time points showed no significant RT changes. DMEK-ME significantly impaired BSCVA (0.38 ± 0.92 logMAR) only 1 month after surgery in comparison to the non DMEK-ME eyes (0.23 ± 0.87 logMAR, *P* = 0.015).

**Conclusion:**

Excluding systemic and surgery-related risk factors, rebubbling increases the risk of DMEK-ME. Performing a CSF scan 1 month after surgery, particularly in rebubbled eyes, efficiently detects DMEK-ME and allows the prompt initiation of treatment, e.g., topical corticosteroid and non-steroidal (NSAID) eye drops. 
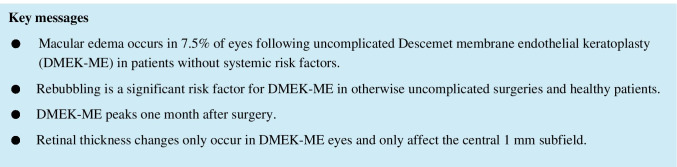

## Introduction

Endothelial keratoplasty has become the gold standard treatment for corneal endothelial dysfunction, as it combines several major advantages over penetrating keratoplasty, including a quicker visual recovery, superior postoperative refractive outcomes, decreased rates of rejection, and an increased postoperative wound strength [1, 2]. Compared with Descemet stripping automated endothelial keratoplasty (DSAEK), Descemet membrane endothelial keratoplasty (DMEK) can produce even better visual acuity results [3–7]. In DMEK, Descemet membrane (DM) and corneal endothelium are transplanted as a treatment for corneal endothelial disorders such as Fuchs endothelial dystrophy (FED) or bullous keratopathy (BK) [8]. The technique was firstly described by Melles et al. in 2006 [9]. Over the years, modifications to the technique were suggested to reduce complications and improve the surgical outcome [10–13].

Common complications of the early postoperative period following DMEK surgery are transplant detachment (4.0–34.6%) [14–16], early postoperative failure (1.4–5.0%) [5, 17], and macular edema (DMEK-ME, 1.0–15.6%) [18–23], the latter peaking between 1 and 3 months after surgery [18–21, 23].

Reported risk factors for DMEK-ME are rebubblings for graft detachment, intraoperative iris damage, and short axial length (AXL) [18–20, 23]. Conditions associated with the development of ME such as chronic intraocular inflammation, retinal vein occlusion, or systemic risk factors, e.g., diabetes mellitus, have not yet proven to increase the DMEK-ME risk [18–20, 23]. Most studies analyzing the occurrence of DMEK-ME included eyes with local or systemic risk factors and intra- or postoperative complications [8, 18–20, 22–24].

In this study, we investigated the occurrence of DMEK-ME following uncomplicated DMEK surgery with neither intraoperative complications, nor systemic or ME-associated eye diseases. Additionally, we evaluated retinal thickness (RT) alterations in various segments of the foveal and parafoveal region by spectral-domain optical coherence tomography (SD-OCT) as well as changes in the best spectacle-corrected visual acuity (BSCVA).

## Materials and methods

### Study type

This retrospective single-center study was conducted by the Department of Ophthalmology, RWTH Aachen University.

### Patient characteristics

The study included 107 eyes of 74 patients undergoing sole DMEK surgery (no combination with other procedures) between 2016 and 2019. The mean age at the time of surgery was 74.5 ± 7.6 (52.1–86.7) years. Forty-one were female, and 33 were male patients. Seventy patients were Caucasian, four were Asian. Surgery was performed on 56 right and 51 left eyes. BSCVA was measured using the Snellen visual acuity chart, and we analyzed results using logarithm of the minimum angle of resolution (logMAR) equivalent units. BSCVA for all patients was 0.75 ± 0.85 (2–0.4) logMAR prior to surgery. The mean AXL measured by optical biometry (IOLMaster 500, Carl Zeiss Meditec AG, Jena, Germany) was 23.21 ± 1.34 (20.68–25.34) mm. Eighty-five eyes underwent DMEK because of FED, 22 for BK. All patients were pseudophakic. All patients had uncomplicated phacoemulsification with in the bag posterior intraocular lens implantation at least 3 months prior to DMEK surgery. Thirty-seven eyes received rebubblings. Five out of 37 received two rebubblings. No eyes received more than two rebubblings.

**Fig. 1:**
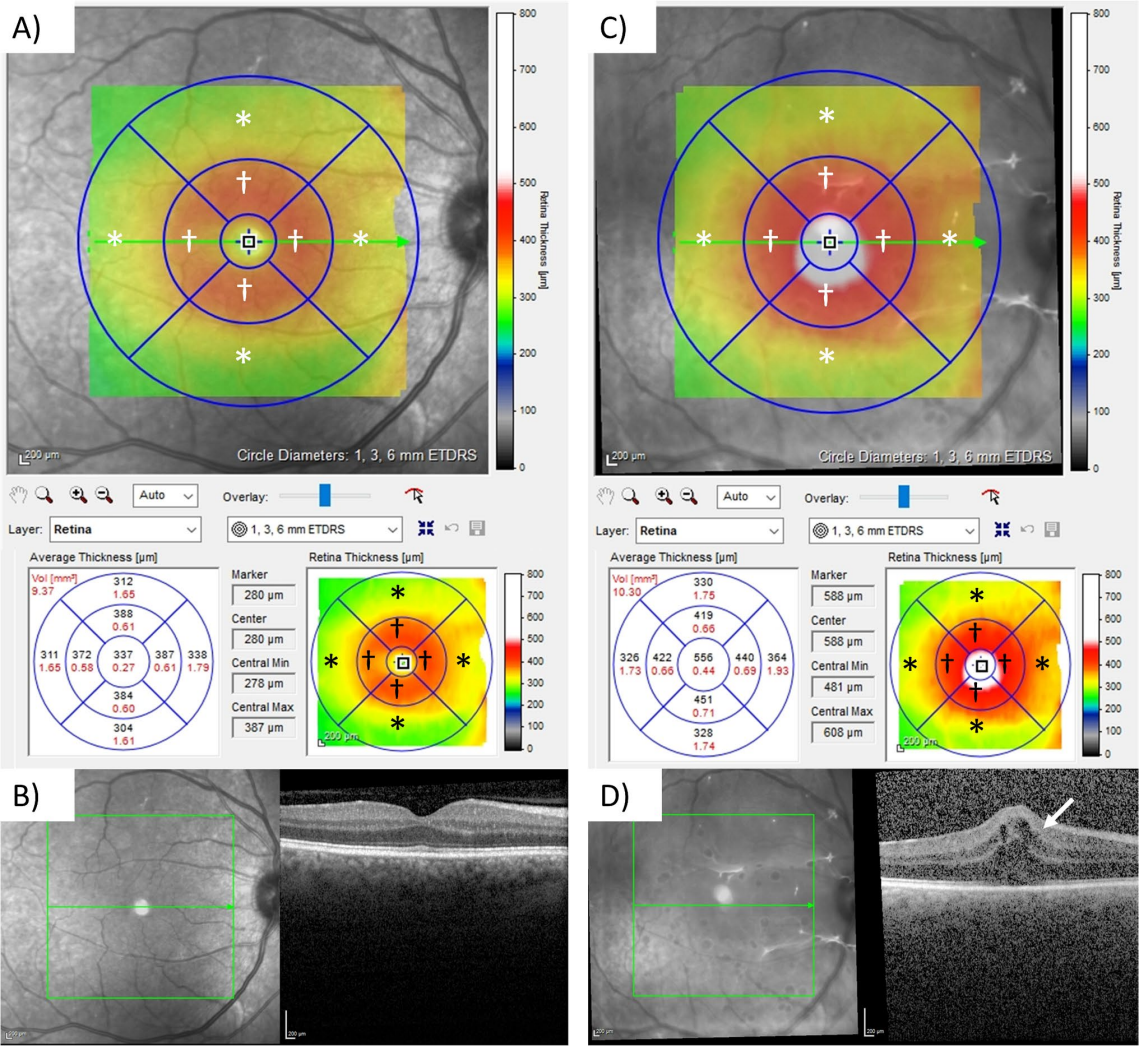
Retinal thickness (RT) map in the HRA/Spectralis Viewing Module (Heidelberg Eye Explorer, Heidelberg Engineering, Heidelberg, Germany) and spectral-domain optical coherence tomography (SD-OCT) imaging prior to uncomplicated Descemet membrane endothelial keratoplasty (DMEK) surgery and 1 month after the surgery with the presentation of macular edema (DMEK-ME). **A** RT map prior to DMEK surgery. **B** *En-face* infrared imaging and SD-OCT imaging of the retina prior to DMEK surgery. **C** RT map one month after DMEK surgery with DMEK-ME. **D** *En-face* infrared imaging and SD-OCT imaging of the retina one month after DMEK surgery. The arrow points at the cystoid macular edema. □ = central subfield (CSF), corresponding to the 1 mm Early Treatment Diabetic Retinopathy Study (EDTRS) subfield. † = 3 mm ETDRS subfield. * = 6 mm ETDRS subfield

DMEK-ME was defined as newly developed subretinal fluid or intraretinal cystoid fluid spaces in the fovea and parafoveal region seen by SD-OCT (Fig. 1; Spectralis-OCT, Heidelberg Engineering GmbH, Heidelberg, Germany).

### Inclusion criteria

Included were pseudophakic patients undergoing uncomplicated sole DMEK surgery without any intraoperative (e.g., iris damage, bleeding or additional intraoperative iridectomies) or postoperative complications (e.g., intraocular pressure (IOP) spikes, pronounced intraocular inflammation and/or fibrin deposition). A rebubbling for transplant detachment was not considered a complication.

### Exclusion criteria

Excluded were eyes with additional eye diseases apart from FED or BK, particularly macular pathologies (e.g., age-related macular degeneration, epiretinal membrane), a known history of ME, diabetic retinopathy (Early Treatment Diabetic Retinopathy Study (ETDRS) criteria) [25, 26], retinal vascular occlusions, chronic intraocular inflammation, and primary or secondary glaucoma. Eyes with any prior ocular surgery apart from cataract surgery were also excluded. Patients with a known history of diabetes mellitus, immunological disorders, or systemic use of anti-inflammatory or immunomodulatory medication (e.g., systemic glucocorticoid therapy) were also excluded. The eyes received no preoperative eye drops apart from lubricating and/or antibiotic eye drops in the case of progressed BK.

### Surgical technique

Two yttrium-aluminum-garnet laser (Visulas YAG II, Carl Zeiss Meditec AG, Jena, Germany) iridotomies were performed inferiorly at least 24 h prior to DMEK surgery. No intraoperative iridectomies were performed. All transplants were pre-stripped 1 day prior to surgery. DMEK surgery was performed as previously described by Melles et al. [9, 10]. All DMEK surgeries were performed under general anesthesia. The donor grafts had a median diameter of 8.0 mm (range: 7.25–8.75 mm). The central host DM was stripped under air aiming for a diameter approximately 1 mm larger than the donor graft. After buffered saline solution exchange (BSS, Alcon, Fort Worth, USA), the stained (trypan blue, VisionBlue, DORC, Rotterdam, Netherlands) donor graft was injected into the anterior chamber (AC). By carefully impressing and tapping the corneal surface with a shallow AC all grafts could be unfolded. An air bubble was injected behind the graft to fixate it. The AC was then fully filled with air. The IOP was estimated and set to normal levels by palpation. In the case of low IOPs, additional air was injected, in high IOPs air released. Finally, a contact lens was placed and dexamethasone-dihydrogen-phosphate disodium 1.0 mg/ml and gentamicin-sulfate 5.0 mg/ml eye drops (Dexa-Gentamicin, Ursapharm, Saarbrücken, Germany) and pilocarpine hydrochloride 20.0 mg/ml eye drops (Pilomann 2%, Bausch Lomb, Rochester, USA) were applied.

The intraocular surgery duration was measured, starting after the preoperative stripping and staining of the donor DM graft with the first paracentesis and ending with the placement of the contact lens at the end of surgery.

Rebubbling was performed in supine position under local anesthesia, when more than one third of the graft was detached between 1 and 4 weeks after DMEK surgery. Following a 23G paracentesis 20% sulfur hexafloride (SF_6_) gas (Arceole pure SF_6_, Arcadophta, Toulouse, France) was injected, aiming for an approx. 90% AC fill. The IOP was checked and a contact lens placed, followed by dexamethasone-dihydrogen-phosphate disodium 1.0 mg/ml, gentamicin-sulfate 5.0 mg/ml, and pilocarpine 20.0 mg/ml eye drops.

### Medication

For the first week after DMEK surgery, patients received dexamethasone-dihydrogen-phosphate disodium 1.0 mg/ml and gentamicin-sulfate 5.0 mg/ml eye drops five times daily and prednisolone acetate 10.0 mg/ml eye drops (Inflanefran forte, Allergan, Dublin, Ireland), five times daily. Pilocarpine hydrochloride 20.0 mg/ml eye drops were applied preoperatively and twice daily during the time of gas in the AC after surgery to induce miosis and reduce the contact of gas to the anterior surface of the IOL, which has been associated with calcium phosphate depositions, particularly in hydrophilic IOLs [27–30]. After the first month, only prednisolone acetate 10.0 mg/ml eye drops were continued. These were tapered by one drop every month to a maintenance dose of once daily.

In case of DMEK-ME nepafenac 1.0 mg/ml eye drops (Nevanac 1 mg/ml, Novartis, Basel, Switzerland) three times daily was added. Nepafenac was continued until complete resolution of DMEK-ME, then tapered by one drop weekly. All DMEK-MEs responded to topical treatment; no further treatment was needed.

### Examinations and follow-up

Eye examinations prior to surgery, 1 week, 1 month, and 6 months after surgery were analyzed. This study evaluated RT measurements via SD-OCT, as well as changes in BSCVA during the 6 months follow-up. The RT was measured in the foveal 1 mm (CSF), parafoveal 3 mm and 6 mm subfield, as defined by the ETDRS research group (1, 3, and 6 mm ETDRS Thickness Map, HRA/Spectralis Viewing Module, Heidelberg Eye Explorer, Heidelberg Engineering, Heidelberg, Germany) [25]. The RT was determined by measuring the distance between inner limiting membrane (ILM) and retinal pigment epithelium (RPE). Figure 1 shows the RT measurement prior to DMEK surgery and 1 month after surgery with the presentation of a DMEK-ME. Additionally, the nasal, superior, temporal, and inferior segment in both the 3 mm (†; Fig. 1) and 6 mm subfield (*; Fig. 1) were analyzed.

### Statistics

If not otherwise specified, all values were expressed as the mean ± standard deviation (range min-max). Statistical analysis was performed using the Statistical Package for the Social Sciences (IBM Corp. Release 2013. IBM SPSS Statistics for Windows, Version 22.0. IBM Corporation, Armonk, New York, USA). Comparisons between categorical variables were conducted using the Fisher’s exact test. For continuous measures the paired and unpaired t-tests were used. According to Kolmogorov–Smirnov tests, all parameters were identified as normally distributed. A *P* value of <0.05 was considered statistically significant.

## Results

During the six months follow-up, eight of 107 eyes (7.5%) developed DMEK-ME. All DMEK-ME were firstly detected during the one-month follow-up. All DMEK-ME showed intraretinal cystoid fluid spaces (Fig. 1), three eyes showed additional subretinal fluid.

No significant difference in age (*P* = 0.073), sex (*P* > 0.999), race (*P* > 0.999), BSCVA prior to surgery (*P* = 0.244), AXL (*P* = 0.395), indication for DMEK surgery (*P* > 0.999), or surgery duration (*P* = 0.629) comparing patients with and without DMEK-ME was observed (Table 1).

**Table 1:** Characteristics of patients, that did or did not develop macular edema following uncomplicated Descemet membrane endothelial keratoplasty

Characteristics	DMEK-ME (*N* = 8/107 (7.4%))	Non DMEK-ME (*N* = 99/107 (92.5%))	*P* value
Age (years)	70.9 ± 6.8 (63.9-83.3)	75.0 ± 7.6 (52.1-86.7)	0.073
Sex			
Male Female	4 (50%) 4 (50%)	44 (44.4%) 55 (55.5%)	>0.999
Race:			
Caucasian Asian	8 (100%) 0	95 (96%) 4 (4%)	>0.999
Right eye Left eye	4 (50%) 4 (50%)	52 (52.5%) 47 (47.5%)	>0.999
BSCVA (logMAR)	0.69 ± 0.87 (2-0.4)	0.77 ± 0.86 (2-0.4)	0.244
AXL (mm)	23.31 ± 0.65 (22.64-24.65)	23.16 ± 1.58 (20.68-25.34)	0.395
Indication:			
FED BK	7 (87.5%) 1 (12.5%)	78 (78.8%) 21 (21.2%)	>0.999
Surgery duration* (minutes)	37.0 ± 10.4 (21-58)	34.3 ± 13.6 (14-80)	0.629
Rebubbling	6 (75%)	31 (31.3%)	0.02
Time to rebubbling (days)	10.8 ± 3.6 (7-16)	12.2 ± 7.4 (2-28)	0.302

*DMEK-ME* macular edema after uncomplicated Descemet membrane endothelial keratoplasty, *AXL* axial length, *BSCVA* best spectacle-corrected visual acuity, *logMAR* logarithm of minimal angle of resolution, *FED* Fuchs endothelial keratopathy, *BK* bullous keratopathy; * surgery duration was measured from the beginning of host Descemet membrane stripping until attachment was achieved by the final air bubble

Six of the eight DMEK-ME eyes (75.0%) had a single rebubbling compared with 31 of 99 eyes (31.3%) not developing DMEK-ME (*P* = 0.02; Table 1). The time to rebubbling was not significantly different (10.8 ± 3.6 (7–16) days in DMEK-ME vs.12.2 ± 7.4 (2–28) days in non DMEK-ME; *P* = 0.302; Table 1). None of the six DMEK-ME eyes receiving a rebubbling needed a second rebubbling, while five of 31 (16.2%) non DMEK-ME eyes received a second rebubbling (*P* = 0.567). In both groups, none of the eyes received more than two rebubblings.

**Fig. 2:**
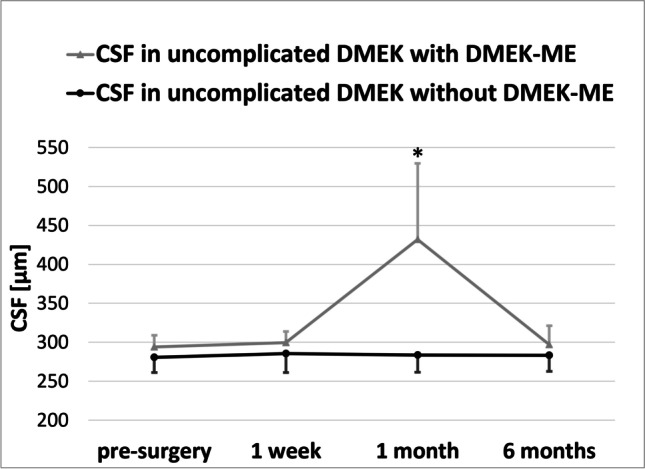
Central subfield (CSF) in μm in uncomplicated Descemet membrane endothelial keratoplasty surgery with and without macular edema at various time points. CSF = central subfield, corresponding to the retinal thickness in the 1 mm subfield of the Early Treatment Diabetic Retinopathy Study (EDTRS) in the retinal thickness map of the HRA/Spectralis Viewing Module (Heidelberg Eye Explorer, Heidelberg Engineering, Heidelberg, Germany). DMEK = Descemet membrane endothelial keratoplasty; DMEK-ME = macular edema after uncomplicated DMEK surgery. * = the CSF in DMEK-ME eyes was significantly thicker one month after surgery compared to pre-surgery and compared to the CSF in non DMEK-ME eyes one month after surgery; *P* < 0.05

During the six months follow-up, neither significant changes in the CSF nor in the 3 mm or the 6 mm subfield were detected in non DMEK-ME eyes (Table 2, Fig. 2).

**Table 2:** Retinal thickness in μm and best spectacle-corrected visual acuity in logMAR at various time points in uncomplicated Descemet membrane endothelial keratoplasty without macular edema and uncomplicated Descemet membrane endothelial keratoplasty with macular edema

		Pre-surgery	1 week postop	1 month postop	6 months postop
RT in DMEK without DMEK-ME [in μm]	CSF	280.8 ± 19.8 (251-315)	285.0 ± 24.2 (241-320)	283.7 ± 22.2 (235-314)	283.3 ± 20.8 (235-315)
	3 mm subfield	339.2 ± 13.3 (322.75-365.25)	344.8 ± 12.8 (327-365.75)	348.6 ± 16.1 (327-375.5)	343.0 ± 16.5 (318.75-376)
	6 mm subfield	295.8 ± 11.2 (280-309)	301.9 ± 13.1 (276.5-315.75)	303.3 ± 13.0 (276.5-318.5)	300.3 ± 14.3 (273-318.75)
BSCVA in DMEK without DMEK-ME [in logMAR]		0.77 ± 0.86 (2-0.4)	0.6 ± 0.74 (1.3-0.22)	0.23 ± 0.87 (0.4-0.0)	0.13 ± 0.72 (0.4-0.0)
RT in DMEK-ME [in μm]	CSF	294.0 ± 14.9 (271-313) *P* = 0.058	299.7 ± 14.3 (276-311) *P* = 0.086	432.0 ± 97.6 * (355-583) *P* = 0.01	297.5 ± 24.3 (264-325) *P* = 0.129
	3 mm subfield	342.3 ± 13.0 (320-361.3) *P* = 0.355	330.4 ± 11.6 (306.5-344.25) *P* = 0.064	384.0 ± 41.4 (350-447.5) *P* = 0.06	348.2 ± 28.8 (276-311) *P* = 0.365
	6 mm subfield	299.2 ± 8.2 (288-314) *P* = 0.287	292.6 ± 5.0 (286.5-297.5) *P* = 0.094	317.0 ± 19.7 (290.5-340.25) *P* = 0.109	300.8 ± 17.0 (275.0-324.25) *P* = 0.480
BSCVA in DMEK-ME [in logMAR]		0.69 ± 0.87 (2-0.4) *P* = 0.244	0.7 ± 1.1 (1.3-0.52) *P* = 0.139	0.38 ± 0.92 (0.7-0.3) * *P* = 0.015	0.14 ± 0.69 (0.4-0.0) *P* = 0.397

*RT* retinal thickness, *DMEK* Descemet membrane endothelial keratoplasty, *DMEK-ME* macular edema after uncomplicated Descemet membrane endothelial keratoplasty. *CSF* central subfield, corresponding to the 1 mm Early Treatment Diabetic Retinopathy Study (EDTRS) subfield of the retinal thickness map of the HRA/Spectralis Viewing Module (Heidelberg Eye Explorer, Heidelberg Engineering, Heidelberg, Germany); 3 mm subfield = 3 mm ETDRS subfield; 6 mm subfield = 6 mm ETDRS subfield. *BSCVA* best spectacle-corrected visual acuity, *logMAR* logarithm of minimal angle of resolution

RT and BSCVA in DMEK-ME compared to RT in DMEK without DMEK-ME. * = *P* < 0.05

**Fig. 3:**
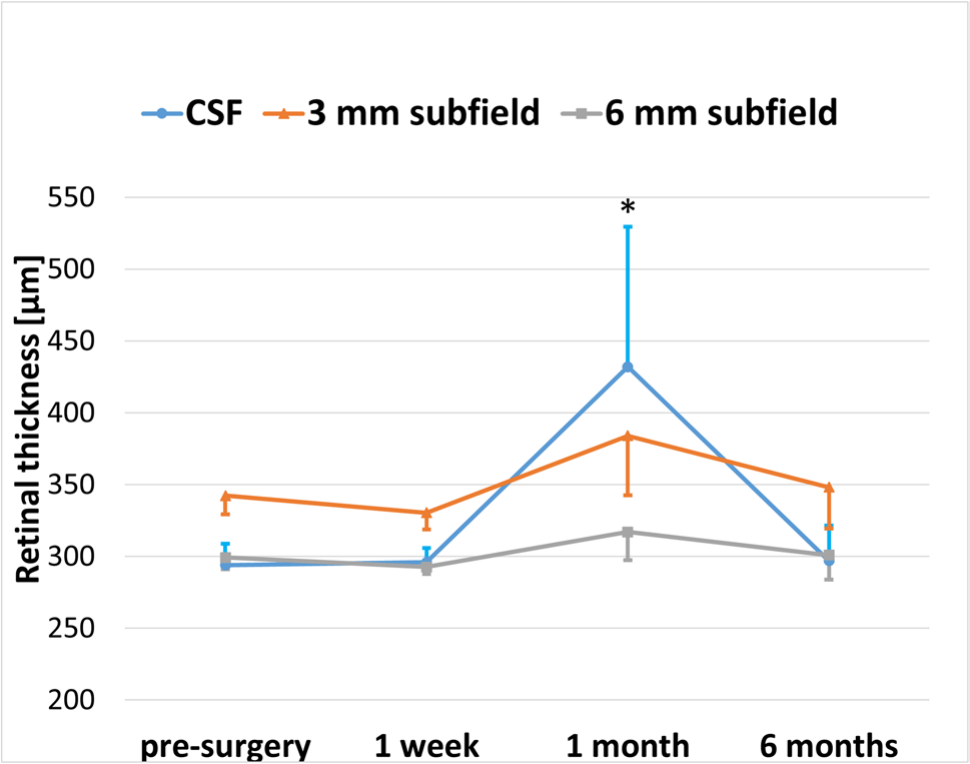
Retinal thickness in μm in uncomplicated Descemet membrane endothelial keratoplasty surgeries developing macular edema (DMEK-ME) in the 1 mm, 3 mm and 6 mm subfield at various time points. CSF = central subfield, corresponding to the retinal thickness in the 1 mm subfield of the Early Treatment Diabetic Retinopathy Study (EDTRS) in the retinal thickness map of the HRA/Spectralis Viewing Module (Heidelberg Eye Explorer, Heidelberg Engineering, Heidelberg, Germany).; 3 mm subfield = 3 mm ETDRS subfield; 6 mm subfield = 6 mm ETDRS subfield. * = the CSF was significantly thicker one month after surgery compared to pre-surgery; *P* < 0.05

In DMEK-ME eyes, the CSF was significantly thicker 1 month after surgery (432.0 ± 97.6 (355–583) μm) compared with before surgery (294.0 ± 14.9 (271–313) μm; *P* = 0.013; Table 2, Fig. 2). Six months after surgery, the CSF had returned to values comparable to before surgery (297.5 ± 24.3 (264-325) μm; *P* = 0.395; Table 2, Fig. 2). The 3-mm and 6-mm subfields showed no significant RT alterations during the follow-up (Table 2, Fig. 3).

Comparing non DMEK-ME and DMEK-ME eyes, the CSF was significantly thicker only one month after surgery (283.7 ± 22.2 (235–314) μm vs. 432.0 ± 97.6 (355–583) μm, *P* = 0.01; Table 2, Fig. 2). Neither the 3-mm nor the 6-mm subfield RT differed significantly between groups during the 6 months follow-up (Table 2).

The analysis of the individual nasal, superior, temporal, and inferior segments of the 3-mm (†; Fig. 1) and 6-mm (*; Fig. 1) subfields found no significant changes comparing DMEK-ME and non DMEK-ME eyes.

BSCVA did not differ between the DMEK-ME and non DMEK-ME group before, 1 week and 6 months after surgery (Table 2). At the 1-month follow-up, the BSCVA was significantly better in the non DMEK-ME group (*P* = 0.015; Table 2).

## Discussion

In this study of 107 eyes undergoing uncomplicated DMEK surgery, eight eyes developed DMEK-ME (7.5%). Omitting known intra- and perioperative risk factors associated with the development of DMEK-ME, rebubbling remained a significant risk factor. DMEK-ME was detected 1 month after surgery, with a significant increase in RT only in the CSF. In non DMEK-ME eyes, no significant changes in the RT occurred. BSCVA was impaired by DMEK-ME but returned to normal after ME resolution at the 6-month follow-up.

Our findings agree with previous studies that reported an incidence of DMEK-ME between 1.0 and 15.6% [18–23]. A wide range of DMEK-ME rates may be caused by varying inclusion criteria in the cited studies. A common exclusion criterion was a history of ME, yet patients with pre-existing risk factors for ME such as diabetes mellitus, a history of uveitis or retinal vein occlusion were partially included [18, 20, 23, 31]. However, the specific effects of these known ME risk factors on DMEK-ME have not been investigated yet [18, 20, 23]. Also, intraoperative complications were frequently not reported in detail and may have been included [18, 20, 22, 23]. In a study on 135 consecutive eyes receiving DMEK, Dapena et al. described a much lower incidence of DMEK-ME of only 0.7% [8]. However, they did not include SD-OCT as a standard examination during follow-up, possibly missing cases of DMEK-ME [8].

In our study, all eight DMEK-MEs were detected 1 month after surgery with no detectable RT increment 1 week after surgery. Similar latencies in the development of DMEK-ME peaking at 1–3 months postoperatively were reported previously [18, 20, 23].

In our study 34.6% of eyes received a rebubbling because of a partial graft detachment. This matches described rebubbling rates of 19.1–66.7% after air tamponade [32]. In a meta-analysis on AC tamponades in DMEK surgery, Marques et al. reported a significantly lower rebubbling rate after 20% SF_6_ tamponade (15.2 vs. 49.0% after air), corresponding to a rebubbling risk reduction by 58.0% [32]. These findings led us to adopt 20% SF_6_ tamponade for all DMEK surgeries from 2019 onwards.

The fraction of our eyes needing a rebubbling was significantly higher in the DMEK-ME compared with the non DMEK-ME group. Inoda et al. used multivariable analysis to similarly show an increased risk for DMEK-ME after rebubbling [20]. In their study, four of twelve patients (33.0%) with DMEK-ME received a rebubbling, compared with nine of 65 (13.8%) without DMEK-ME [20]. They speculated that rebubbling causes mechanical stress on the iris leading to subclinical inflammation and DMEK-ME [20]. Interestingly, Heinzelmann et al. did not find an increased risk for DMEK-ME after rebubbling, stating that repeated rebubblings may only cause minor stress on the ocular tissue [23]. They described that 14 of 80 (18.0%) patients needed a rebbubling after sole DMEK surgery, while 17 of 75 (23.0%) needed a rebubbling after Triple-DMEK [23]. A proportional hazards Cox model showed no correlation between rebubblings and DMEK-ME [23]. Kocaba et al. did not report a significant correlation between rebubblings and DMEK-ME either [18]. They reported that ~60% of patients undergoing DMEK surgery needed a rebubbling, yet neither in sole DMEK nor Triple-DMEK surgery rebubblings were a risk factor for DMEK-ME [18]. The opposing findings for rebubblings as a risk factor for DMEK-ME could be due to several reasons. Some studies partially included patients suffering from reported risk factors for ME such as diabetes mellitus, history of uveitis, or retinal vessel occlusion [18, 20, 23, 31]. Others did not clearly state the time of rebubbling [18, 23]. Only Inoda et al. reported that all 13 rebubblings occurred within 7 days after surgery [20]. Most importantly none of the discussed studies specified the volume of gas injected into the AC during rebubbling, which could have led to differences in the induced iris stretch and inflammation [18, 20, 23]. We aim for an almost complete AC fill (approx. 90%), potentially inducing more iris stretch than other groups using lower volumes. Nevertheless, an exact quantification of the injected intracameral gas volume remains a challenge in clinical practice.

As our study intentionally excludes other reasons for the development of DMEK-ME our findings support Inoda’s hypothesis that iris stretch, and a resulting subclinical inflammation can lead to DMEK-ME [20]. Hoerster et al. implicated an inflammatory cause of DMEK-ME by showing a significant reduction in DMEK-ME rates after increasing topical corticosteroids after DMEK surgery from five times daily to hourly for the first week after surgery [19]. Inoda et al. described iris damage as a significant risk factor for DMEK-ME in sole and staged (phacoemulsification and intraocular lens implantation exactly one month prior to DMEK) DMEK surgery as another leading cause for the development of DMEK-ME [20]. In our study, uncomplicated sole DMEK surgery was performed, excluding cases of intraoperative iris damage and additional inflammatory stress.

Several studies on the development of ME after retinal vein occlusion and in diabetic eyes showed a reduction of risk for edema development in the case of vitreous detachment [33]. Three mechanisms were discussed: (1) An attached vitreous exerts tangential forces on the retina resulting in an increased production of growth hormones and proinflammatory mediators [34–36]. (2) The attached vitreous may serve as a reservoir for proinflammatory mediators and growth factors [34, 37]. (3) A detached vitreous improves retinal oxygenation due to a higher diffusion rate from the anterior segment [38]. Higher retinal oxygenation constricts retinal arterioles and thereby decreases hydrostatic pressure which counteracts the development of retinal edema [38]. To our knowledge the effect of vitreous detachment particularly on the development of DMEK-ME has not been evaluated yet. Larger cohort studies could investigate this topic in the future. However, the study design might be challenging as corneal diseases and corneal edema can limit the OCT quality and therefore make a reliable evaluation of the posterior vitreous difficult. Focusing on early stages of FED and BK cases could be a viable option.

In the literature a wide range of topical, oral, subconjunctival and intravitreal treatments for DMEK-ME have been described [18, 20, 23]. In our study, all DMEK-MEs (*n* = 8) responded to the combination of topical corticosteroid and non-steroidal anti-inflammatory (NSAID) treatment. In case of DMEK-ME, Inoda et al. similarly added NSAID eye drops (bromfenac sodium sesquihydrate 1.0 mg/ml) twice daily and applied a single subconjunctival triamcinolone acetonide injection (undisclosed dose) [20]. With this treatment regime, they saw the full resolution of all DMEK-MEs (*n* = 12) [20]. However, the duration of treatment was not specified [20]. Kocaba et al. treated all DMEK-ME (*n* = 11) patients with 250 mg acetazolamide orally three times a day for 2 months [18]. The patients received either additional topical corticosteroid (dexamethasone 1.0 mg/ml) or NSAID (indomethacin 1.0 mg/ml) eye drops three times daily for 2 months. However, how the patients were allocated to the groups was not described [18]. This treatment regime led to the resolution of DMEK-ME in ten of eleven patients during the 2 months of treatment [18]. The one patient with a persistent DMEK-ME received an intravitreal corticosteroid implant (dexamethasone 0.7 mg) [18]. In this case, the treatment’s effect on the DMEK-ME was not addressed [18]. Heinzelmann et al. treated DMEK-ME patients (*n* = 20) with a combination of topical corticosteroid (prednisolone acetate 10.0 mg/ml) and NSAID eye drops (ketorolac-trometamol 5.0 mg/ml) four times daily and 125 mg acetazolamide orally twice daily for 6 weeks [23]. In 19 patients this treatment regime led to the full resolution of DMEK-ME during the 6 weeks of treatment [23]. One patient with persistent DMEK-ME received additional intravitreal injections of bevacizumab and triamcinolone [23]. The dose, number, and time of treatment were not reported [23]. The DMEK-ME resolved under the intravitreal therapy, yet the time to full resolution was not specified [23]. First-line topical corticosteroid and NSAID treatment might have been effective in treating DMEK-ME in our study because we excluded all patients with intra- and postoperative complications and possible risk factors for ME. To date studies comparing the efficiency of different DMEK-ME treatments are still lacking, and there is no agreement on a standardized approach [18, 20, 23].

The surgery duration of our patients matched time spans previously reported by Heinzelmann et al. for sole DMEK surgery (31.0 min) [23]. We did not find a difference in surgery duration between DMEK-ME and non DMEK-ME eyes (37.0 vs. 34.3 min). Surgery duration was not specified in other publications on DMEK-ME either [18–22]. However, we would expect an increasing incidence of DMEK-ME with longer surgery time, as this is usually associated with intraoperative complications and augmented inflammation [39].

Shorter AXL has previously been described as a potential risk factor for DMEK-ME [23]. The hypothesis is that in shorter eyes, proinflammatory cytokines from the anterior segment reach higher concentrations in the foveal region [23]. We did not find a significant difference in AXL comparing DMEK-ME and non DMEK-ME eyes, yet the sample size and the AXL range in our study did not allow a subgroup analysis comparing the DMEK-ME risk in short compared with long eyes. Similarly, Inoda et al. previously investigated this question without detecting a relationship [20]. With a sample size of 77 eyes, a subgroup analysis on significantly longer or shorter eyes was not performed in their study either [20].

In non DMEK-ME eyes, no significant changes in RT over the 6-month follow-up were observed. This could lead to the assumption that an all-or-nothing mechanism could determine the development of DMEK-ME. Reports on DMEK-ME support this hypothesis by showing a wide margin between the RT in DMEK-ME and non DMEK-ME eyes (542.0 vs. 244.0 μm [20]; 507.0 vs. 262.0 μm [19]; our study: 432.0 vs. 283.7 μm) suggesting a jump in mean RT rather than a gradual increase between groups.

In this study, we did not include Triple-DMEK surgery. Previous studies comparing sole to Triple-DMEK surgery neither found differences in the DMEK-ME rates nor significant RT changes in the non DMEK-ME eyes from before to after surgery [18, 22, 23]. However, they did not look at the different RT subfields [18, 22, 23].

Interestingly, while not for DMEK, gradual RT elevations have been described for other anterior segment surgeries. In combined cataract and DSAEK surgery, Mashor et al. showed an increase in macular RT after 1 month, even if no ME was detected [40]. However, combined cataract and DSEAK surgery are considered a more invasive procedure with larger corneal cuts being performed leading to pronounced postoperative inflammation [41, 42].

To our knowledge, this is the first study evaluating RT changes not only in the CSF but also the 3-mm and 6-mm subfields, as well as their nasal, superior, temporal, and inferior sub-segments. Compared with eyes without DMEK-ME, the RT in eyes developing DMEK-ME showed an increase only 1 month after surgery and only in the CSF. There was no significant difference, neither in the 3 mm nor the 6 mm subfield leading to the assumption that the retinal alterations occurring during DMEK-ME focus on the CSF. To detect DMEK-ME, RT measurements should concentrate on this area. In our study, the RT in the CSF in DMEK-ME was 432.0 μm, which matched the reported CSF thickness in DMEK-ME in other studies (401.0–542.0 μm) [18–20, 23]. The CSF values measured before surgery and at the 6-month follow-up in both our groups agree with previously reported measurements in healthy eyes [43–45]. However, CSF measurements are known to vary depending on race, age, devices, and software versions [43–47]. This must be considered when comparing RT values of different studies.

The BSCVA of our patients that did not develop a DMEK-ME improved quickly after surgery and reached values of 0.23 logMAR (approx. 0.6 decimal) at the 1-month follow-up and 0.13 logMAR (approx. 0.8 decimal) at the 6-month follow-up. This swift and excellent visual recovery has previously been described for DMEK [48–51]. With 0.38 logMAR (approx. 0.4 decimal), the BSCVA of our DMEK-ME group was significantly inferior at the 1-month follow-up. DMEK-ME has previously been reported to interfere with visual acuity [19, 23]. Hoerster et al. described a decrease in the best corrected visual acuity (BCVA) by 0.15 logMAR during DMEK-ME [19]. Heinzelmann et al. similarly showed worse BSCVA results in DMEK-ME compared with non DMEK-ME eyes (approx. 0.5 vs. 0.2 logMAR) [23].

In our study, with RT returning to normal values at the 6-month follow-up, the BSCVA recovered to match the non DMEK-ME group. The full visual recovery in eyes after resolution of DMEK-ME has previously been reported [18, 20, 21]. Inoda et al. found no significant difference in postoperative BSCVA after 6 months in eyes with and without DMEK-ME (0.12 vs. 0.07 logMAR) [20]. Kocaba et al. found no differences in BSCVA in patients with and without DMEK-ME after 6-month follow-up either (0.3 vs 0.3 logMAR) [18]. However, due to recruiting a wider range of patients, the overall BSCVA after DMEK was worse compared with our data. In a study by Flanary et al. all patients with resolved DMEK-ME reached a BSCVA of 0.2 logMAR or better, and ~ 70% reached a BSCVA of 0.1 logMAR or better at the 6-month follow-up [21].

As a limitation of our study, due to organizational reasons, we did not examine our patients 3 months after surgery. Kocaba et al. stated that the highest incidence of DMEK-ME was seen between 1 and 3 months after surgery, yet most studies report the highest incidence one month after surgery [18, 20, 23].

In conclusion, in uncomplicated DMEK surgery when excluding systemic and surgery-related risk factors, rebubbling remains an important risk factor for DMEK-ME. To detect DMEK-ME, we recommend performing a CSF measurement 1 month after DMEK surgery, particularly in rebubbled eyes, as they showed higher rates of DMEK-ME. Due to the shown latency, CSF scans 1 month after surgery are most efficient in detecting DMEK-ME and allow the prompt initiation of treatment. In this study all DMEK-MEs resolved with topical corticosteroid and NSAID eye drops.

## Data Availability

Not applicable.
